# Radiotherapy planning in a prostate cancer phantom model with intraprostatic dominant lesions using stereotactic body radiotherapy with volumetric modulated arcs and a simultaneous integrated boost

**DOI:** 10.3389/fonc.2023.1147593

**Published:** 2023-04-28

**Authors:** Agnieszka Skrobala, Marta Kruszyna-Mochalska, Kinga Graczyk, Adam Ryczkowski, Magdalena Fundowicz, Piotr Milecki, Julian Malicki

**Affiliations:** ^1^ Department of Electroradiology, Poznan University of Medical Science, Poznan, Poland; ^2^ Department of Medical Physics, Greater Poland Cancer Centre, Poznan, Poland; ^3^ Department of Radiation Oncology I, Greater Poland Cancer Centre, Poznan, Poland

**Keywords:** SBRT-SIB, DIL, VMAT, transit and non-transit dosimetry, patient-specific quality assurance, phantom model

## Abstract

**Aim:**

In the treatment of prostate cancer with radiation therapy, the addition of a simultaneous integrated boost (SIB) to the dominant intraprostatic lesions (DIL) may improve local control. In this study, we aimed to determine the optimal radiation strategy in a phantom model of prostate cancer using volumetric modulated arc therapy for stereotactic body radiotherapy (SBRT-VMAT) with a SIB of 1-4 DILs.

**Methods:**

We designed and printed a three-dimensional anthropomorphic phantom pelvis to simulate individual patient structures, including the prostate gland. A total of 36.25 Gy (SBRT) was delivered to the whole prostate. The DILs were irradiated with four different doses (40, 45, 47.5, and 50 Gy) to assess the influence of different SIB doses on dose distribution. The doses were calculated, verified, and measured using both transit and non-transit dosimetry for patient-specific quality assurance using a phantom model.

**Results:**

The dose coverage met protocol requirements for all targets. However, the dose was close to violating risk constraints to the rectum when four DILs were treated simultaneously or when the DILs were located in the posterior segments of the prostate. All verification plans passed the assumed tolerance criteria.

**Conclusions:**

Moderate dose escalation up to 45 Gy seems appropriate in cases with DILs located in posterior prostate segments or if there are three or more DILs located in other segments.

## Introduction

In recent years, the development of advanced radiation therapy techniques such as volumetric modulated arc therapy (VMAT) and stereotactic body radiation therapy (SBRT) has improved long-term cancer outcomes. The precision of these advanced modalities allows for dose escalation as high as 75.6 Gy in prostate cancer (PCa) (1). However, dose escalation is limited by the risk of toxicity to surrounding healthy tissues (2). The American Society for Radiation Oncology (ASTRO) recommends high-dose SBRT for select low- and intermediate-risk PCa patients (3, 4). For ultrahypofractionated schemes, the recommended strategies are 35 Gy (five fractions of 7 Gy) or 36.25 Gy (5 fr x 7.25 Gy). Although higher doses (> 50 Gy in 5 fractions) to the entire prostate yield excellent 5-year biochemical control rates, there is an increased risk of high-grade toxicity (5, 6).

Due to the risks associated with high-dose SBRT to the whole prostate, an alternative approach is to selectively increase the dose to the dominant intraprostatic lesion (DIL), which can be identified using multiparametric T2-weighted, dynamic contrast-enhanced and/or diffusion-weighted prostate magnetic resonance imaging (MRI) (7). This approach is highly promising, especially considering that more than 70% of men with newly-diagnosed PCa have a confirmed DIL > 0.5 cm^3^ (8, 9). Studies have shown that treating the dominant lesion through a targeted boost dose significantly improves treatment outcomes while reducing doses to healthy surrounding tissues (10). This approach is particularly interesting given that these intraprostatic lesions are the most common site of local failure (11).

Several different options are available to deliver the boost dose to the DIL. Some small studies have shown that the boost can be delivered using conventional fractionation or moderate hypofractionation to the whole prostate and the focal lesion (6, 12–18). An alternative approach is to deliver a simultaneous integrated boost (SIB) to the DIL using SBRT (SBRT-SIB). Although this technique is not widely used at present, it has been shown to improve the therapeutic ratio and may improve local control, in addition to shortening the course of radiotherapy (19–21).

The SBRT dose is normally prescribed according to ASTRO recommendations (36.25 Gy in 5 fractions), but there are no strict rules regarding the dose to the DILs. In the studies performed to date, the mean dose to the DIL was > 47 Gy (19–21). A prospective pilot study evaluated 36.25 Gy in 5 fractions to the whole prostate with a SIB of 40 Gy to the DILs, showing that this scheme was feasible, within the accepted dosimetric constraints to the organs-at-risk (OAR), and would result in acceptable acute morbidity (21). However, the optimal SIB dose to the DIL has not yet been clearly established.

In this context, the aim of the present study was to determine the optimal SIB dose to the DIL within dose-volume constraints. More specifically, we sought to determine the feasibility of planning and irradiating up to four DILs in an anthropomorphic phantom model of PCa using SBRT-VMAT. We also evaluated the influence of different SIB doses (40, 45, 47.5, and 50 Gy) and the number and location of the DILs within the prostate gland on dose distribution.

## Material and methods

### Anthropomorphic pelvis phantom

**Figure 1:**
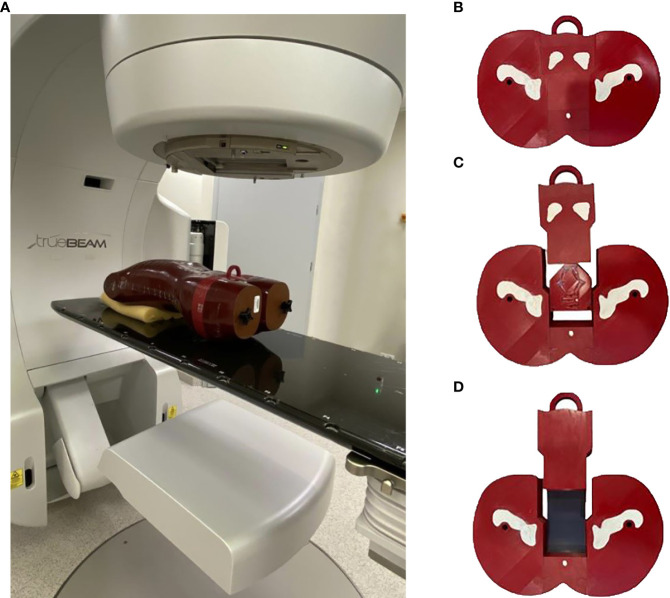
**(A)** The modified anthropomorphic pelvis phantom was combined to form the 3D printing of the entire prostate region in the irradiated position. **(B)** The four parts of the 3D printed, modified anthropomorphic pelvis phantom. **(C)** Axial section of the phantom with four dominant intraprostatic lesions. **(D)** Axial section of the phantom with film inserts.

This study was carried out in a modified anthropomorphic Alderson-RANDO phantom. The two horizontal slices in the anthropomorphic pelvis were combined to print the entire prostate in three dimensions (3D) (**Figure 1A**). This 3D printed piece was based on tissue maps created on a 3D computed tomography (CT) scan of the phantom. The mesh was processed for 3D printing using Blender software (blender.org). To export G-code 3D printer instructions for a fused deposition, we used the 3D modeling printer Prusa i3 MK3S+ Prusa Slicer software (Prusa Research, Prague, Czech).

Printing was performed (90% infill; 0.2 mm per layer) with an aliphatic linear polyester (PLA) filament (FiberLab S.A., Poland). The percentage of infill filament was selected to match Hounsfield units (HU) values from the original slice of the anthropomorphic phantom close to water, ranging from 0 HU to 25 HU (mean, 10 HU). This phantom consists of pelvic bones (gypsum) and soft tissues to simulate the prostate, bladder, rectum, and other tissues. Gypsum was used to simulate bone parts, resulting in a mean HU value of 560 (range, 460 - 580 HU). To simulate the DILs, a 1.5 ml Eppendorf flask was used and filled with water. The Eppendorf flask was used to visualise the DILs on CT scans and for radiobiological assessment.

The phantom was printed in four different sections rather than a single piece. This was due, in part, to the limitations of the 3D printer but also to allow for the insertion of DILs and radiochromic films (**Figure 1B**). Four metal fiducial markers were embedded in the prostate region to allow for verification of geometric positioning during irradiation. Two male pelvises were printed separately, one to calculate the dose distribution with four Eppendorf flasks (**Figure 1C**) and the second to perform the measurements (**Figure 1D**). The phantom slice was cut along the two axial planes, thereby creating a slot to insert a single piece of film to measure doses throughout both the prostate and rectum, and a second film to measure doses in the bladder region (**Figure 1D**).

### Contouring

The realistic clinical target volume (CTV) included the whole prostate and organs at risk (OAR), including the bladder, rectum, and femoral heads were contoured. These targets were delineated on the anthropomorphic phantom CT scans to simulate the original patient anatomy. Superficial layers of the phantom body (thickness = 3 mm) were considered skin. The PTV was generated based on the criteria used to establish prostate margins for SBRT at our institution, as follows: a 5 mm margin in all directions around the CTV, with a slightly smaller margin (3 mm) in the posterior direction. This PTV was defined as PTV_(Prostate)_. The margin was established in accordance with previous studies on prostate SBRT (19, 20). We added a uniform 2 mm margin to the CTV_(boost)_ of the DILs (identified by the Eppendorf volume), which we defined as the boost volume, PTV_(boost)_. This volume (i.e, the PTV_(boost)_) was subtracted from the PTV_(Prostate)_.

### Prescribed dose

**Figure 2:**
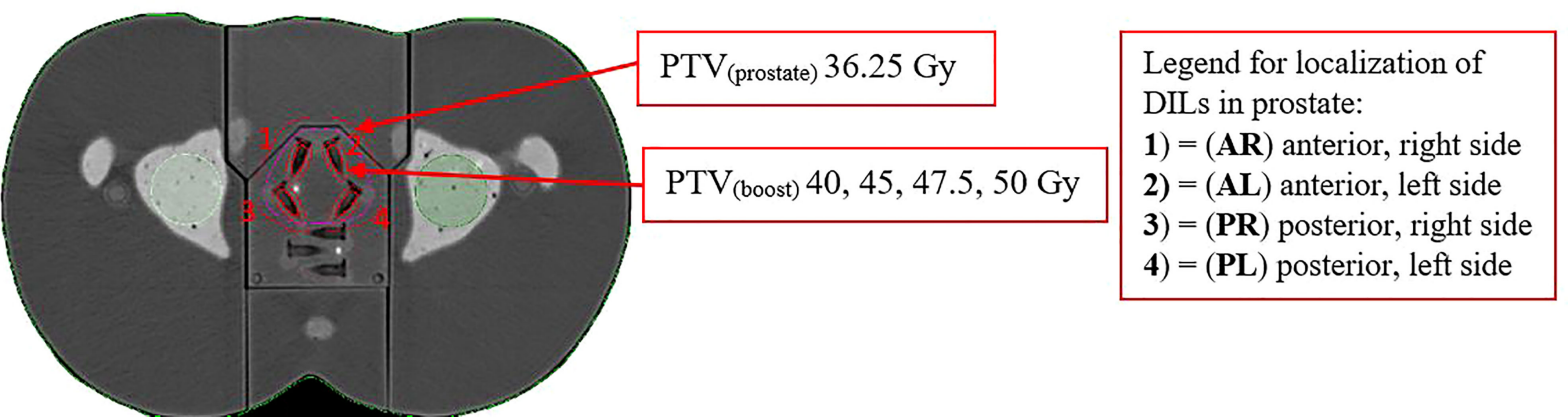
Planning schema of SBRT-SIB PTV_(prostate)_ to the dominant intraprostatic lesions PTV_(boost)_ in the following locations: (1) = (AR) middle anterior, right side, (2) = (AL) middle anterior, left side, (3) = (PR) middle posterior, right side and (4) = (PL) middle posterior, left side. The figure shows an axial slice of the planning CT scan in the phantom model in which the target and organ at risk volumes are contoured. The data for this plan were transferred from a patient’s CT scan.

The planned dose was 36.25 Gy in five fractions to the whole prostate PTV_(Prostate)_ in accordance with ASTRO guidelines (3). Four different SIB doses [PTV_(boost)_] were delivered to the intraprostatic lesions (40, 45, 47.5, and 50 Gy) (19–21). The DILs were located in four different segments of the prostate, resulting in 15 different possible configurations (dose and DIL localization), as follows; (1) middle anterior, right side (AR); (2) middle anterior, left side (AL); (3) middle posterior, right side (PR); and (4) middle posterior, left side (PL) (as shown in **Figure 2**). For these possible configurations, we evaluated irradiation of a single DIL (4 configurations), two DILs (6 configurations), three DILs (5 configurations), and four DILs (1 configuration). Consequently, there were 15 different possible DIL configurations at four different doses, resulting in a total of 60 treatment plans.

The plan aimed to cover 95% of the PTV_(boost)_ with 95% of the SIB dose, and 100% of the SIB dose with ≥ 98% of CTV_(boost)_. To meet these criteria, all plans were normalized such that ≥ 95% of the PTV_(Prostate)_ received 100% of the prescribed dose. No limits on target dose heterogeneity were specified, but the plans were designed to avoid exceeding the maximum dose (D_max_), defined as 120% of the prescribed dose to the PTV_(Prostate)_. Guidelines for treatment planning dosimetry goals for the target volume, evaluated parameters, and formulas are presented in **Table 1**. The dose constraints to the OARs were based mainly on normal tissue dose constraints for SBRT described by Hanna et al. (2018) as shown in **Table 2** (22). Urethra usually was identified on the multi-parametric MRI or defined as the outer contour of the Foley catheter, so it would not be feasible to define it in the regiment of phantom study.

**Table 1:** Treatment plan dosimetric goals for target volume parameters and formulas applied.

Target volume	Parameter	Prescription or formula
DIL = CTV_(boost)_	V_95%_	100% of PD_boost_ to 95% CTV_(boost)_
PTV_(boost)_	V_98%_	100% of PD_boost_ to 98% PTV_(boost)_
Whole prostate = CTV_(Prostate)_	V_95%_	100% of PD to 95% CTV_(Prostate)_
PTV_(Prostate)_	V_95%_	100% of PD to 95% PTV_(Prostate)_
	D_max_	< 120%
	CI	≤ 1.5; CI= V_95%_/PTV
	HI	No limits; HI=D_max_/PD
	GI	No limits; GI=PIV_50%_/PIV
Monitor units	MU	No limits

DIL, dominant intraprostatic lesion; CTV, clinical target volume; PTV, planning target volume; V, volume; PD_boost_, boost prescription dose; PD, prescription dose; D_max_, maximum dose; CI, conformity index; HI, homogeneity index; GI, gradient index; PIV prescribed isodose volume.

**Table 2:** OAR planning constraints for SBRT-SIB plans based primarily on normal tissue dose constraints for stereotactic radiotherapy defined by Hanna et al. (22).

Organ at risk	Constraints	
Rectum	V 36Gy < 1cm^3^
	V 36.25Gy < 5%
	V 32.60Gy < 10%
	V 29Gy < 20%
	V 18Gy < 50%
Bladder	V 37Gy < 10cm^3^
	V 36.25Gy < 10%
	V 32.60Gy < 15%
	V 29Gy < 25%
	V 18Gy < 55%
Femoral heads	V 18Gy < 4 cm^3^
Skin	D_max_ < 15Gy

### Treatment planning for the boost to the dominant intraprostatic lesions

The dose distribution was calculated using VMAT plans prepared in the Varian Eclipse treatment planning system ver. 16.1 (TPS; Varian Medical Systems, Palo Alto, CA, United States [USA]). SBRT-SIB calculations were prepared for a total of 60 treatment plans (15 DIL configurations at four different doses). The dose calculation was prepared using the anisotropic analytical algorithm (AAA) with a grid size of 0.125 cm. Plans were generated for a 10 MV flattening filter-free (FFF) beam using two full arcs on a Varian TrueBeam linear accelerator.

Doses were calculated, verified, and measured using transit and non-transit dosimetry for patient-specific quality assurance (QA).

### Non-transit and transit and dosimetry for dose delivery verification

To detect clinically relevant errors in the radiation delivery, the accuracy of dose deliverability was assessed by performing dosimetric verification based on measurement and calculation techniques of all 60 plans. A secondary dose calculation was performed using the DoseCHECK module of the SunCheck platform (Sun Nuclear, Melbourne, FL, USA). Dose delivery verification was performed at two time points in the radiotherapy process, before (non-transit dosimetry) and during (transit dosimetry) treatment.

Before treatment, pre-verification (non-transit) was performed using two different tools: 1) 2D-EPID-based absolute dosimetry (called “Fraction 0”), which was performed with the SunCheck platform module (PerFRACTION) (Sun Nuclear, Melbourne, FL, USA) and 2) OCTAVIUS-4D Rotational Phantom with suitable OCTAVIUS-4D Detector array 1600 SRS (PTW, Freiburg, Germany).

*In vivo* dose verification during irradiation in the phantom model was performed using 2D-EPID-based transit dosimetry (called “Fraction N”) using the same SunCheck platform module (PerFRACTION). Simultaneously, Gafchromic EBT-XD and EBT3 films (Ashland, Wayne, NJ, USA) were used to verify the plans with the highest and lowest total doses. For verification purposes (to compare TPS calculations and measured doses), image guidance was performed before dose delivery using cone beam CT to confirm the position of the anthropomorphic phantom and to ensure alignment with the fiducial markers.

The Suncheck web-based platform was also used, thus enabling quality control with a dose calculator for an independent calculation (DoseCheck) and both fractions 0 and N. The dose calculation algorithms used in this software are model based, with collapsed cone algorithm heterogeneity correction and dose calculation by a superposition/convolution (S/C) technique.

The 1600 SRS array consists of 1521 liquid-filled ionization chambers organized in a grid over a 15×15 cm^2^ area. In the center area (6.5 x 6.5 cm), detector spacing is 2.5 mm. This array was inserted into the OCTAVIUS-4D cylindrical polystyrene phantom, which can rotate synchronously with the gantry, thus enabling 3D dose reconstruction. The 1600 SRS array was calibrated by a cross-calibration factor for the equivalent field size to the quadratic field against the Semiflex 31002 ionization chamber to ensure dose measurements independent of the TPS. A Phyton-developed script was used to determine the equivalent field size for each plan, based on the RT plan in DICOM format, determined for specific control points. Verisoft software, v. 8.0 (PTW, Freiburg, Germany) was used to compare the planned and measured dose distribution.

Gafchromic films were used to measure radiation in the anthropomorphic pelvis through film inserts specially designed for this study (**Figure 1**). EBT-XD films were used to verify dose distributions for the highest doses (~ 10 Gy); for lower doses, EBT3 films were used. Calibrations were also acquired before QA to slightly scale the film dose as required for a range of doses that are measurable by both film types. Film.tiff files, obtained by scanning with an EPSON Perfection v850 Pro scanner (Seiko Epson Corporation, Japan), were processed with the FilmQA Pro software (Ashland, Wayne, NJ, USA) using a dose calibration curve and the triple channel method.

All gamma index analyses were performed by absolute dose comparison with a global or local γ calculation and a 10%/5% low dose cut‐off threshold. Global normalization was used as this is considered more relevant than local normalization for QA of clinical cases. In addition, local analysis was performed to account for the detailed differences between the implementation of the plans. Dosimetric verification was judged optimal if the percentage of points fulfilling gamma index criteria exceeded 95%, using a global criterion (DD) of 2% and a distance-to-agreement (DTA) of 3 mm with a 10% threshold (non-transit, Fraction 0) in accordance with the recent recommendations of the AAPM TG 218 (23). Based on clinical experience at our institution for independent calculation, pre-verification with OCTAVIUS-4D and Fraction 0, we narrowed our criteria, as follows: local dose criterion was DD 2% and DTA was 2 mm with a 10% threshold (23). For the film and transit dosimetry (Fraction N), the following criteria were applied: DTA, 3 mm; DD, 3%; and global approach with a 10% threshold; the plan was accepted as correct when the passing rate was ≥ 90%.

### Statistical analysis

A Kruskal-Wallis analysis of variance (ANOVA) was performed, with adjusted p-values of 0.05 indicating statistical significance.

## Results

The four DILs were located in four of the twelve prostate segments in the phantom model, as follows: DIL (1) = (AR) anterior right; DIL (2) = (AL) anterior left, DIL (3) = (PR): posterior right, and DIL (4) = (PL) posterior left (**Figure 2**). The treatment volumes for each DIL were as follows: CTV_boost_ 0.6 cc and PTV_boost_ 1.7 cc. The volume of DILs 2, 3, and 4 (irradiated simultaneously) plus a 2 mm margin were 3.5, 5.4, and 7.2 cc, respectively. OAR volumes were as follows: bladder, 131 cc; rectum, 37.1 cc; right and left femoral heads, 24.4 cc and 28.4 cc, respectively.

Dose planning parameters and dose delivery verifications were evaluated according to the SIB dose (40, 45, 47.5, and 50 Gy) and the location of the DIL relative to certain OARs to determine how this affected dose distribution (evaluation by prescribed dose to the DIL).

The first set of measurements was performed according to the SIB dose (40, 45, 47.5, 50 Gy) (evaluation by DIL prescribed dose). The plans were denominated as follows: SIB_40Gy, SIB_45Gy, SIB_47.5Gy, and SIB_50Gy plans. There were 15 plans in each group.

The second set of measurements was performed according to the location the DIL to the nearest, most important OAR: the bladder or rectum or the bladder and rectum (evaluation by DIL location in prostate segments). The treatment plans were labelled according to the location of the DIL (posterior vs. anterior), as follows: SIB_nB (anterior segment, near bladder), SIB_nR (posterior near rectum), and SIB_nBR (anterior and posterior near both bladder and rectum). There were 20 plans in each group.

### Treatment planning dosimetry for the boost to the dominant intraprostatic lesions

**Figure 3:**
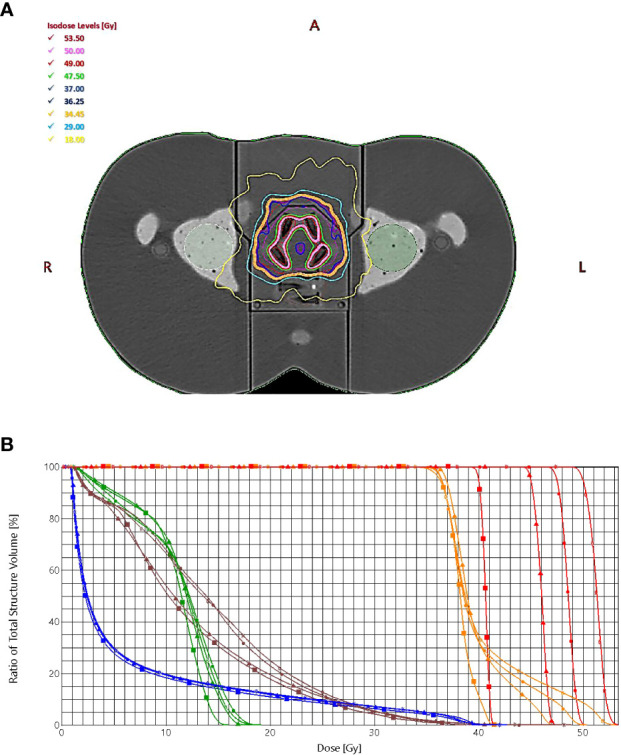
**(A)** The dose distribution for plans with four simultaneously irradiated DILs with doses up to 50 Gy. **(B)** The dose-volume histogram of the target (PTV_prostate_ = orange, PTV_boost_ = red) and organs at risk (bladder = blue, rectum = brown, left femoral head = green), prescribed dose (squares = 40 Gy, triangles = 45 Gy, circles = 47.5 Gy, stars = 50 Gy).

The treatment plans met all protocol-defined goals for all cases. **Figure 3** shows the dose distribution for plans with four simultaneously irradiated DILs (up to 50 Gy) (**Figure 3A**), and the dose-volume histogram (DVH) of the same four DILs with all four SIB doses (40, 45, 47.5 and 50 Gy) (**Figure 3B**).

**Table 2** shows the results of the SBRT-SIB planning study by prescribed dose to the DILs. All target volumes (CTV_boost_, PTV_boost_, CTV_prostate_, PTV_prostate_) achieved the planning goal (i.e., met the criteria shown in **Table 1**).

The maximum point dose ranged from 103% to 109% of the PTV_boost_ volume for each plan. Doses to all 15 possible DIL configurations for all four doses were obtained. The doses to the OARs (bladder, rectum, femoral heads and skin) fulfilled the constraint criteria given in **Table 2**. The prescribed doses (40, 45, 47.5, and 50 Gy) for the DILs in the various SBRT-SIB plans are shown in the last five columns of **Table 3**. For most parameters, the SIB dose had only a negligible impact on OAR constraints, except for certain parameters directly influenced by the prescribed dose, such as the maximum dose to the PTV_boos_ and the homogeneity index. However, when comparing higher dose SBRT-SIB plans (45 vs. 50 Gy), the dose had a significant influence on some of the rectal constraints (V 29 Gy < 20% and V18 Gy < 50%) For the bladder, two constraints (V 37Gy < 10cc and V 36.25Gy < 10%) showed statistically significant differences: an increase in the prescribed dose led to an increase in the volume of the OARs for doses > 45 Gy (**Table 3** and **Figure 3A**). Similarly, the number of monitor units increased in line with the increase in SIB dose (**Table 3**); however, there were no statistically significant differences.

**Table 3:** Comparison of treatment planning dosimetry goals for the target volume: CTV and PTV and OARs planning constraints used for the SBRT-SIB plans according to prescribed dose to the DILs (40Gy, 45Gy, 47.5Gy, and 50Gy).

Structure	Parameter	SBRT-SIB plan (mean+SD) // evalution by the DIL the prescibed dose					Overall	Multiple comparsions					
								40vs.45	40vs.47.5	40vs.50	45vs.47.5	45vs.50	47.5vs.50
		SIB_40Gy	SIB_45Gy	SIB_47.5Gy	SIB_50Gy		p-value	p-value < 0.05					
CTV(boost)	V95%	99.71 ± 0.58	100.00 ± 0.00	99.96 ± 0.14	99.87 ± 0.51		0.185	0.059	0.105	0.428	0.326	0.326	0.49
PTV(boost)	V95%	95.31 ± 0.64	95.07 ± 0.26	95.07 ± 0.26	95.15 ± 0.44		0.391	0.195	0.189	0.453	0.972	0.536	0.52
CTV(Prostate)	V98%	98.70 ± 1.25	99.04 ± 1.88	99.26 ± 1.15	98.89 ± 1.60		0.766	0.565	0.206	0.711	0.692	0.824	0.472
PTV(Prostate)	V95%	96.55 ± 1.33	96.60 ± 1.49	96.98 ± 0.87	97.60 ± 0.91		0.068	0.921	0.3	**0.018**	0.399	**0.036**	0.7
	Dmax	104.05 ± 0.87	105.49 ± 0.88	106.38 ± 1.11	106.91 ± 0.92		**<0.001**	**<0.001**	**<0.001**	**<0.001**	**0.023**	**<0.001**	0.163
	CI	1.15 ± 0.04	1.15 ± 0.04	1.15 ± 0.03	1.18 ± 0.03		0.092	0.669	0.823	0.055	0.787	**0.031**	**0.013**
	HI	1.14 ± 0.01	1.29 ± 0.02	1.37 ± 0.02	1.44 ± 0.03		**<0.001**	**<0.001**	**<0.001**	**<0.001**	**<0.001**	**<0.001**	**<0.001**
	GI	3.18 ± 0.06	3.19 ± 0.04	3.20 ± 0.04	3.21 ± 0.05		0.355	0.6	0.343	0.124	0.642	0.234	0.397
PTV(Prostate) – PTV(boost)	V95%	96.40 ± 1.39	96.46 ± 1.55	96.86 ± 0.88	97.49 ± 0.95		0.069	0.907	0.288	**0.018**	0.396	**0.038**	0.07
Rectum	V 36Gy < 1cc	0.37 ± 0.12	0.36 ± 0.13	0.38 ± 0.06	0.42 ± 0.11		0.494	0.81	0.835	0.28	0.613	0.192	0.231
	V 36.25Gy < 5%	0.89 ± 0.29	0.89 ± 0.33	0.95 ± 0.16	1.03 ± 0.29		0.487	0.988	0.527	0.211	0.551	0.234	0.36
	V 32.60Gy < 10%	2.87 ± 0.69	2.61 ± 0.54	2.61 ± 0.31	3.02 ± 0.51		0.099	0.262	0.195	0.502	0.997	**0.042**	0.013
	V 29Gy < 20%	5.91 ± 0.83	5.50 ± 0.61	5.58 ± 0.59	6.27 ± 0.82		**0.019**	0.138	0.219	0.234	0.727	**0.001**	**0.012**
	V 18Gy < 50%	23.66 ± 1.64	22.98 ± 0.97	23.61 ± 2.56	26.09 ± 3.48		**0.004**	0.861	0.999	**0.033**	0.888	**0.004**	**0.029**
Bladder	V 37Gy < 10cc	4.11 ± 0.76	4.27 ± 0.57	4.42 ± 0.29	4.65 ± 0.30		**0.041**	0.516	0.155	**0.016**	0.39	**0.031**	**0.038**
	V 36.25Gy < 10%	3.78 ± 0.40	3.76 ± 0.42	3.84 ± 0.24	4.02 ± 0.24		0.141	0.935	0.57	0.051	0.542	**0.048**	**0.048**
	V 32.60Gy < 15%	5.66 ± 0.35	5.54 ± 0.40	5.58 ± 0.30	5.78 ± 0.29		0.235	0.398	0.542	0.302	0.302	0.069	0.077
	V 29Gy < 25%	7.21 ± 0.38	7.06 ± 0.42	7.10 ± 0.35	7.32 ± 0.33		0.217	0.315	0.403	0.393	0.795	0.068	0.08
	V 18Gy < 55%	12.63 ± 0.48	12.44 ± 0.53	12.48 ± 0.47	12.80 ± 0.46		0.185	0.311	0.4	0.342	0.82	0.058	0.074
Femoral head R	V 18Gy < 4 cc	0.00 ± 0.00	0.00 ± 0.00	0.00 ± 0.00	0.00 ± 0.00		0.267	**<0.001**	0.326	0.162	0.326	0.162	0.483
Femoral head L	V 18Gy < 4 cc	0.00 ± 0.00	0.00 ± 0.00	0.01 ± 0.04	0.00 ± 0.02		0.85	0.326	0.326	0.302	0.346	0.346	0.649
Skin	Dmax < 15Gy	10.98 ± 0.61	10.91 ± 0.39	11.35 ± 0.90	11.58 ± 0.81		**0.037**	0.705	0.195	**0.03**	0.091	**0.007**	0.473
Monitor units	MU	2277 (1986-2538)	2280 (2042-2574)	2372 (2100-2879)	2426 (2070-3149)		0.091	0.952	0.15	0.049	0.166	0.055	0.501

The bold numbers indicate significant differences between the plans (ANOVA test).

**Table 4** compares the treatment planning dosimetry goals for the target volume by DIL location. As expected, all target volumes (DIL, prostate, and planning volume) met the planning goals. Even with dose escalation to 50 Gy, the dose to the bladder and rectum were within the stipulated constraints. However, as **Table 4** shows, the plans involving high doses (SIB_50Gy) and DILs located in posterior segments (SIB_nR) were close to violating the constraints.

**Table 4:** Comparison of treatment planning dosimetry goals for the target volume: CTV and PTV and OAR planning constraints used for the SBRT-SIB plans, evaluated by DIL location (near the bladder, near the rectum, and near both the bladder and rectum).

Structure	Parameter	SBRT-SIB plan (mean+SD) // evalution by the DIL localisation			Overall	Multiple comparsions		
						nBvs.nR	nBvs.nBR	nRvs.nBR
		SIB_nB	SIB_nR	SIB_nBR	p-value	p-value < 0.05		
CTV(boost)	V95%	100.00 ± 0.02	99.82 ± 0.56	99.84 ± 0.39	0.31	0.336	0.438	0.981
PTV(boost)	V95%	95.26 ± 0.57	95.05 ± 0.16	95.14 ± 0.41	0.27	0.241	0.622	0.764
CTV(Prostate)	V98%	98.68 ± 1.88	99.07 ± 1.32	99.17 ± 1.16	0.613	0.687	0.549	0.973
PTV(Prostate)	V95%	96.51 ± 1.36	97.41 ± 0.93	96.88 ± 1.24	0.065	0.053	0.589	0.347
	Dmax	105.39 ± 1.45	105.96 ± 1.23	106.78 ± 1.60	0.44	0.421	0.666	0.915
	CI	1.16 ± 0.03	1.16 ± 0.04	1.16 ± 0.04	0.941	0.937	0.992	0.972
	HI	1.31 ± 0.18	1.31 ± 0.11	1.32 ± 0.11	0.978	0.999	0.981	0.983
	GI	3.21 ± 0.05	3.17 ± 0.03	3.21 ± 0.05	**< 0.01**	0.002	0.976	0.003
PTV(Prostate) – PTV(boost)	V95%	96.37 ± 1.40	97.30 ± 0.97	96.74 ± 1.30	0.067	0.055	0.63	0.325
Rectum	V 36Gy < 1cc	0.32 ± 0.08	0.44 ± 0.12	0.39 ± 0.09	**< 0.01**	**< 0.01**	0.05	0.24
	V 36.25Gy < 5%	0.77 ± 0.21	1.09 ± 0.30	0.96 ± 0.20	**< 0.01**	**< 0.01**	0.042	0.164
	V 32.60Gy < 10%	2.60 ± 0.26	2.95 ± 0.68	2.79 ± 0.58	0.131	0.11	0.489	0.643
	V 29Gy < 20%	5.61 ± 0.40	5.93 ± 0.98	5.90 ± 0.80	0.36	0.398	0.468	0.992
	V 18Gy < 50%	23.52 ± 1.22	24.58 ± 3.16	24.21 ± 2.84	0.416	0.393	0.668	0.892
Bladder	V 37Gy < 10cc	4.23 ± 0.62	4.50 ± 0.54	4.36 ± 0.44	0.286	0.254	0.706	0.703
	V 36.25Gy < 10%	3.80 ± 0.29	3.91 ± 0.41	3.83 ± 0.32	0.286	0.254	0.706	0.703
	V 32.60Gy < 15%	5.65 ± 0.25	5.64 ± 0.43	5.63 ± 0.34	0.989	0.997	0.988	0.997
	V 29Gy < 25%	7.20 ± 0.27	7.15 ± 0.46	7.17 ± 0.39	0.918	0.911	0.968	0.984
	V 18Gy < 55%	12.65 ± 0.37	12.52 ± 0.58	12.59 ± 0.53	0.701	0.677	0.91	0.903
Femoral head right	V 18Gy < 4 cc	0.00 ± 0.00	0.00 ± 0.00	0.00 ± 0.00	0.314	0.821	0.625	0.287
Femoral head left	V 18Gy < 4 cc	0.00 ± 0.00	0.00 ± 0.00	0.00 ± 0.03	0.166	0.999	0.218	0.237
Skin	Dmax < 15Gy	11.14 ± 0.60	11.02 ± 0.64	1.47 ± 0.90	0.147	0.985	0.342	0.141
Monitor units	MU	2319 (1988-2597)	2315 (2070-2486)	2382 (2040-3145)	**< 0.01**	**< 0.01**	**< 0.01**	**< 0.01**

The bold numbers indicate a statistically significant difference between plans (ANOVA test).

### Non-transit dosimetry and transit for dose delivery verification

#### The SunCheck platform PerFRACTION: (Fraction 0) and (Fraction N)

**Figure 4:**
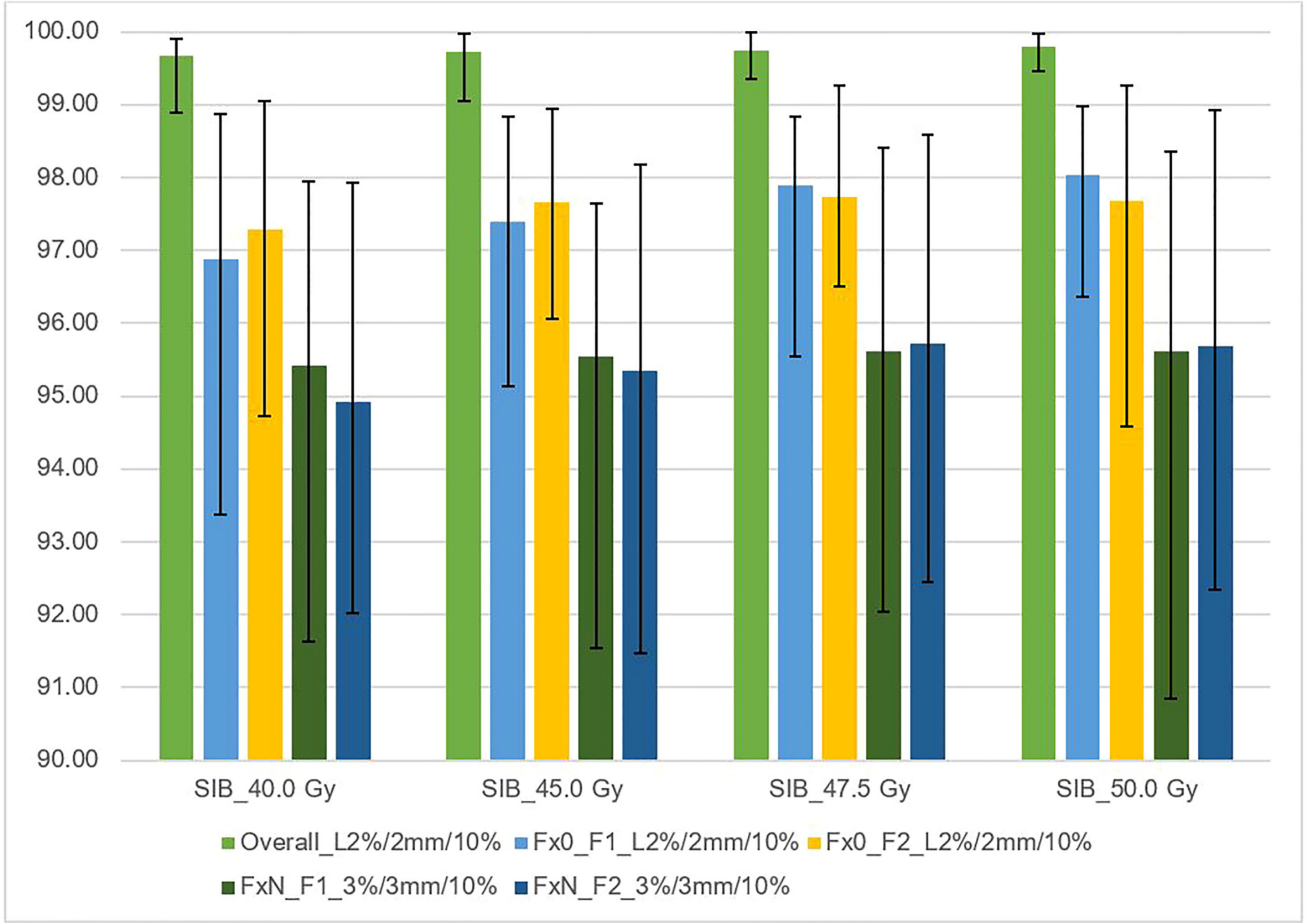
This figure shows the average gamma passing rate [%] vs. total planned dose (SIB_40Gy, SIB_45Gy, SIB_47.5Gy, SIB_50Gy) for three sets of dosimetric methods: 1) overall gamma, local approach with DoseCheck calculation method (DD 2%, DTA 2 mm, TH 10%); 2) Fraction 0, non-transit [Fx0] (local approach, DD 2%, DTA 2 mm, TH 10%), and 3) Fraction N, transit dosimetry [FxN] (global approach DD 3%, DTA 3 mm, TH 10%). Field-by-field dose (F1- field 1, F2- field 2) and composite (for overall gamma). Black bars represent the maximum and minimum passing rates for each result.

**Figure 4** shows the relationships between the gamma passing rates vs. total planned dose with various criteria for independent calculations (DoseCheck), 2D-EPID-based pre-verification (Fraction 0), and transit dosimetry (Fraction N). The mean passing rate for dose delivery verification before treatment (non-transit dosimetry), the rate was ≥ 96.8%. During treatment (transit dosimetry), the passing rate was ≥ 94.9% (accepted criteria ≥ 90%).

**Figure 5:**
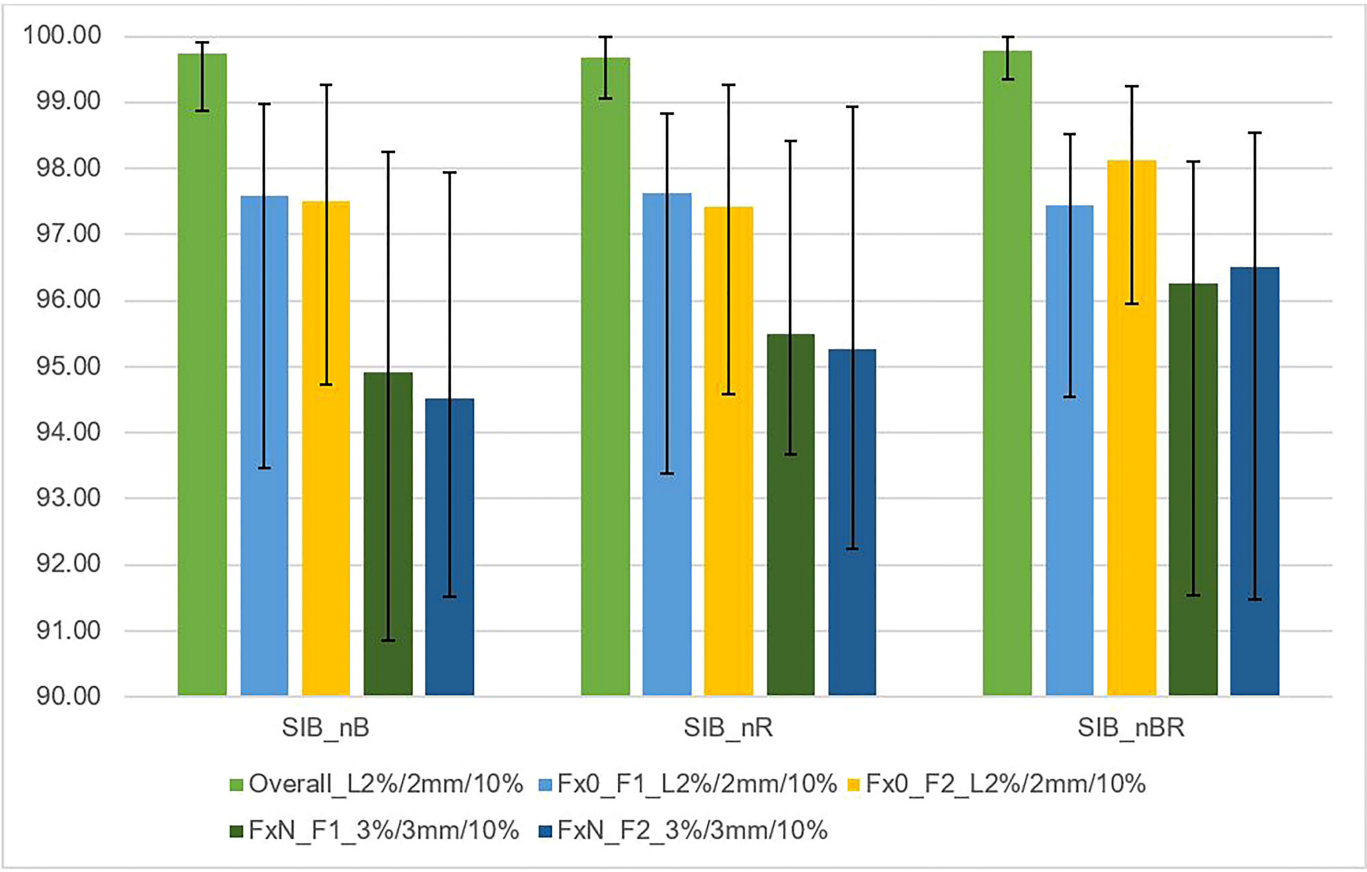
This figure shows the average gamma passing rate [%] vs. DIL location relative to critical organs (SIB_nB, SIB_nR, SIB_nBR) for three sets of dosimetric methods: 1) overall gamma, local approach with DoseCheck calculation method (DD 2%, DTA 2 mm, TH 10%); 2) Fraction 0, non-transit [Fx0] (local approach, DD 2%, DTA 2 mm, TH 10%), and 3) Fraction N, transit dosimetry [FxN] (global approach DD 3%, DTA 3 mm, TH 10%). Field-by-field dose (F1- field 1, F2- field 2) and composite (for overall gamma). Black bars represent the maximum and minimum passing rate values for each result.

**Figure 5** shows the relationship between the gamma passing rate versus the DIL location relative to critical organs with various gamma criteria for independent calculation (DoseCheck), 2D-EPID-based pre-verification (Fraction 0), and transit dosimetry (Fraction N). The passing rate was > 94.5% for all plans. For dose delivery verification before treatment (non-transit dosimetry), the passing rate was ≥ 97.3%. During treatment (transit dosimetry), the passing rate was ≥ 94.5% (accepted criteria ≥ 90%).

#### OCTAVIUS-4D rotational phantom with the OCTAVIUS detector 1600 SRS

The non-transit dosimetry results were obtained from the measurement performed with the OCTAVIUS-4D phantom using the OCTAVIUS detector array 1600 SRS.

**Figure 6:**
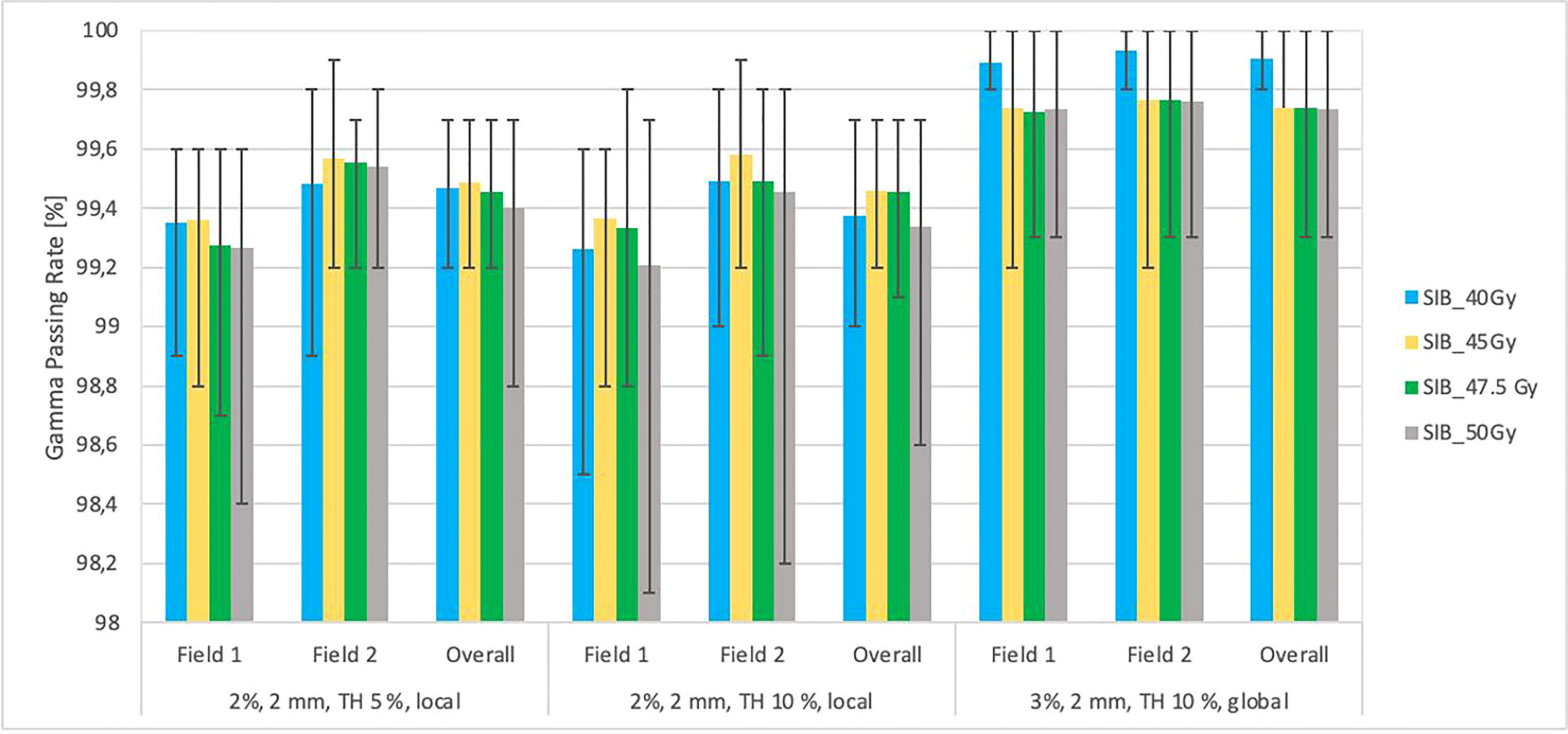
This figure shows the mean gamma passing rate [%] vs. the total planned dose (SIB_40Gy, SIB_45Gy, SIB_47.5Gy, SIB_50Gy) for three sets of testing parameters: local approach, DD 2%, DTA 2 mm, TH 5%; local approach, DD 2%, DTA 2 mm, TH 10%; DD 3%, DTA 2 mm, global approach TH 10%. The field-by-field dose and composite with maximum and minimum values are marked by black bars. Field-by-field (F1- field 1, F2- field 2) and composite (for overall gamma) with the maximum and minimum passing rates are marked by black bars. The results were obtained from measurements (non-transit dosimetry) performed in OCTAVIUS-4D Rotational Phantom with the OCTAVIUS Detector 1600 SRS.

**Figure 6** shows the relationship between the gamma passing rates vs. total planned dose with various criteria (DD 2%, DTA 2 mm, TH 10%, local, DD 2%, DTA 2 mm, TH 5%, local, DD 3, DTA 2 mm, TH 10%, global). Each field was analyzed separately, as was the composite distribution of the two fields. All plans had a passing rate > 99.2% (accepted criteria ≥ 95%).

**Figure 7:**
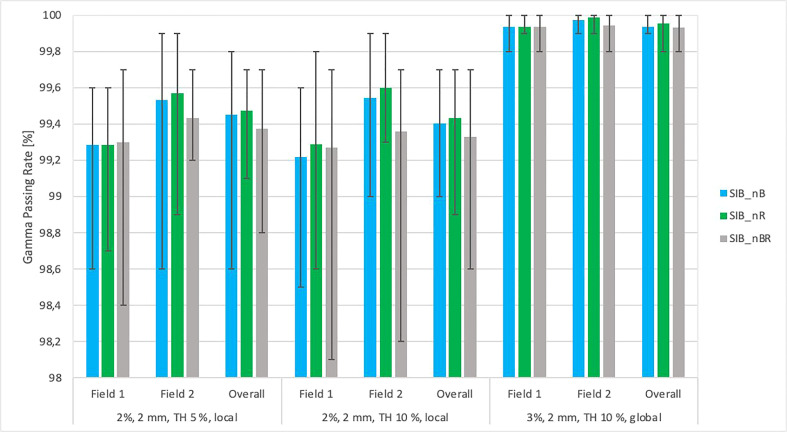
The graph of average gamma passing rate [%] vs. the DIL location relative to critical organs (SIB_nB, SIB_nR, SIB_nBR) for three sets of testing parameters: local approach, DD 2%, DTA 2 mm, TH 5%; local approach, DD 2%, DTA 2 mm, TH 10%; DD 3%, DTA 2 mm, global approach TH 10%. The field-by-field dose and composite with maximum and minimum values are marked by black bars. Field-by-field (F1, field 1; F2, field 2) and composite (for overall gamma) with the maximum and minimum passing rate values are marked by black bars. The results were obtained from measurements (non-transit dosimetry) performed the OCTAVIUS-4D Rotational Phantom with the OCTAVIUS Detector 1600 SRS.

**Figure 7** shows the relationship between the gamma passing rates vs. the location of lesions relative to critical organs using various criteria: DD 2%, DTA 2 mm, TH 10%, local, DD 2%, DTA 2 mm, TH 5%, local, DD 3%, DTA 2 mm, TH 10%, global. Each field was analyzed separately, as was the composite distribution of the two fields. The maximum and minimum values were determined in groups with a specific location relative to critical organs. The results were obtained from measurements (non-transit) in the Octavius-4D phantom with the OCTAVIUS-4D Rotational Phantom with the OCTAVIUS Detector 1600 SRS. All plans had an accepted passing rate > 99.2% (accepted criteria ≥ 95%).

#### Gafchromic EBT3 and EBT-XD in the anthropomorphic phantom pelvis

**Figure 8:**
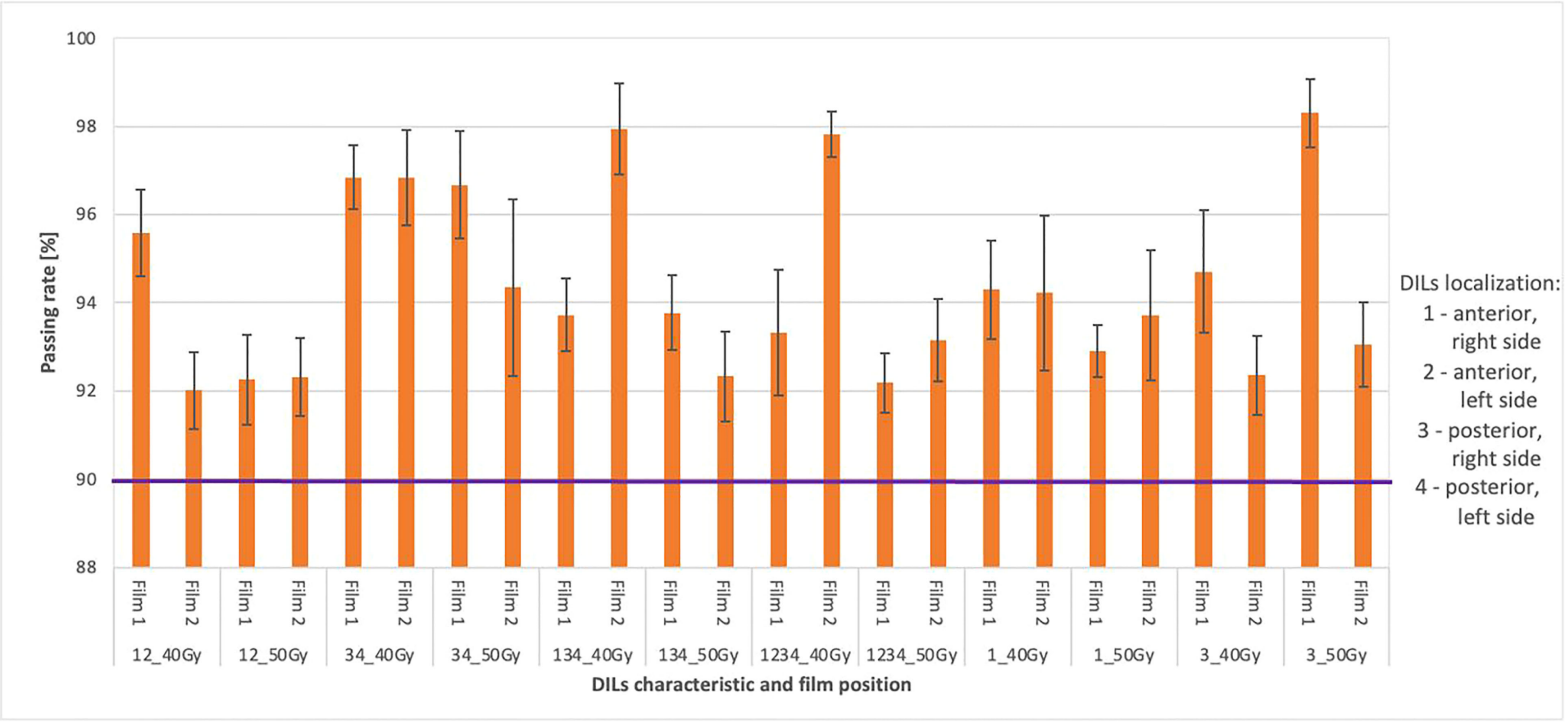
Shows the average gamma passing rate [%] for selected treatment plans using the following gamma testing parameters: DD 3%, DTA 3 mm, TH 10%, global approach. The numbers 1 and 2 indicate doses measured in the prostate and rectum (1) and doses measured in the bladder region (2). The composite dose with the maximum and minimum passing rate values is marked by black bars. The purple colour marks the limit values above which the plan is considered correct.

The relationship between the tiple-channel average gamma passing rates for selected treatment plans with testing criteria (DD 3%, DTA 3 mm, TH 10%, global) are shown in **Figure 8**. The films were placed in the transverse plane of the phantom in the appropriate insert; the first film 1 measured doses in the prostate and rectum; a second film (film 2) measured doses in the bladder region. The composite distribution of the two fields was analyzed. All plans had an accepted passing rate > 92% for dose delivery verification before (transit film dosimetry) treatment (accepted criteria ≥ 90%).

## Discussion

In this study, we sought to determine the optimal plan and delivery strategy for SBRT-SIB for 1-4 DILs in an anthropomorphic phantom model of PCa. The main purpose of this study was to establish the planning strategy of SBRT-SIB for the DIL. As our results show, it is possible to deliver a steep dose gradient to this focal lesion and reduce the dose to the remaining part of the prostate, even for high SIB doses to the focal lesion (50 Gy).

Many studies have compared different planning systems to deliver SBRT-SIB. For example, Tree at al compared RapidArc and MultiPlan while Kim et al. compared different SIB doses delivered by CyberKnife-based plans (5, 19). One study even compared photons with protons Cambria et al. (24). Nevertheless, none of the studies performed to date have compared different escalated doses administered with VMAT and the influence of localization and number of DILs. Our study is the first to do so. We compared the impact of different planning parameters on outcomes, performing both transit and non-transit dose verification dosimetry for the phantom model. Our findings showed that the dose coverage met the protocol requirements for all targets, in line with the findings of other studies (5, 19, 20, 25). Prostate PTV coverage was excellent, with at least 95% of the 36.25 Gy prescription covering the whole prostate PTV in all plans. The boost PTV coverage was also good, with 98% meeting OAR constraints, similar to the findings reported by Kim et al. and McDonald at al (5, 25). However, under certain circumstances, the doses to the OARs increased, reaching levels that were close to violating the constraint parameters to the rectum. This was observed in two situations: 1) when four DILs were treated simultaneously and 2), when the DILs were located in the posterior part of the prostate, in line with the findings reported by Kim et al. and Feng et al. (5, 20).

**Table 2** shows OAR planning constraints for SBRT-SIB plans. We found that SIB doses ≥ 47.5 Gy were close to violating OAR constraints, especially for the rectum (**Table 3**). However, contouring the urethra in the regiment of the phantom study would not be feasible, analysis of restrictions for the urethra was carried out. The best sparing of the urethra was achieved where V100% of the prescribed dose should be less than 50% or the maximum dose in the urethra was less than 120% of the prescribed dose. Summarizing the best sparing of the urethra was achieved by moderate dose escalation up to 45 Gy chosen as appropriate in our study compared. All verification plans passed the assumed tolerance criteria. These findings suggest that moderate dose escalation up to 45 Gy seems appropriate for DILs located in posterior segments or in cases with ≥ 3 DILs. Tree at al (19). and Kim et al. (5) reported similar findings indicating that SBRT-SIB can produce clinically-acceptable treatment plans with a focal boost of 47.5 Gy in 5 fractions; however, in posterior localizations, the dose should be reduced to a maximum of 45 Gy (5, 19).

Dose escalation with SBRT to the DILs could improve the therapeutic ratio while also avoiding the need to administer high doses (> 36.25 Gy) to the prostate gland, which would increase toxicity (10). However, this strategy requires accurate mapping of the dominant focal lesion to the CT image (7, 20). In this regard, dose escalation to the DILs is an appealing approach, as it would allow for higher doses to one or more focal lesions without increasing toxicity. However, at present, the optimal dose to these lesions has not been well-established. Similarly, due to the lack of data, we only partially understand the influence of the number of simultaneously irradiated DILs and the location of these lesions.

In the present study, we used doses as high as 50 Gy, which has been shown to improve local control (26), although such high doses to the entire prostate are associated with an increased risk of high-grade toxicity (5). This study was designed to assess the influence of boost doses up to 50 Gy to focal lesions based on the planning technique used for this study, which can be delivered in the anthropomorphic phantom conditions. However, the distance between the DIL and the rectum, the size of the PTV and OARs, and the patients’ body size and shape can be dramatically impact radiation toxicity, particularly with high doses (e.g., 50 Gy). Tree et al. made a similar observation (i.e., the prescribed SIB dose is correlated with distance to the rectum), even though the maximum dose was slightly lower in their study (47.5 Gy) (19). The SIB dose can be escalated, but we should consider methods for rectal sparing, such as the use of a spacer (27).

We defined the planning strategy and verification plans based on data obtained from a 3D-printed anthropomorphic phantom, in contrast to other studies (5, 21), which did not use a phantom. In our study, the use of an anthropomorphic phantom was needed to ensure precise film placement and to realize transit dosimetry. Given the importance of having a phantom with organs and DILs located in the same locations as a human body, we built a custom, 3D-printed anthropomorphic phantom (**Figure 1**). As **Figure 1** shows, the phantom was easy to assemble, allowing for repositioning of the DILs and film inserts without any discernible displacement. The HU number and the other parameters indicate agreement between the 3D printed section and the RANDO anthropomorphic phantom, similar to the study by Giacometi et al. (28). In that study, in which the 3D printed phantom also enabled transit dosimetry (2%/2mm criteria), gamma analysis was performed to compare TPS calculations to the measured dose when delivering flattening filter free (FFF) SBRT-SIB plans, finding a passing rate of 97.0% versus 95% in our study for 2D-EPID based dosimetry (**Figure 4**) and 92% for film dosimetry (**Figure 8**).

We evaluated a total of 60 different treatment plans resulting from the combination of 4 DILs localizations (**Figure 2**) and various SIB doses (range, 40 to 50 Gy). Even though the target goals and OARs constraints were met in all cases, we found similar to other studies that when the DILs were located in the posterior part of the prostate, there was a substantially higher likelihood that the plan would not meet OAR constraints (5, 20).

We compared a total of 60 different SBRT-SIB plans, with 15 possible configurations (localisation, volume, and numbers of DILs) and four different boost doses. All treatment plans successfully satisfied the protocol-defined goals (**Table 2**) in all cases; thus, the primary feasibility endpoint was met. Prostate PTV coverage was excellent, with 95% of the 36.25 Gy prescription dose covering the entire prostate PTV in all cases and the 98% CTV (boost) volume covering the whole range of dose-escalated doses at the 100% level (**Tables 3**, **4**). Boost coverage of the PTV was good at the 95% level, in which > 96.5% of all plans met OAR constraints (**Tables 3**, **4**).

The plans that came closest to violating the rectal constraints (V36Gy < 1cc) were those in which the DILs were located in posterior segments of the prostate (SIB_nR and SIB_50Gy plans). The 40 Gy treatment plans (SIB_40Gy) yielded values similar to those observed for the 36 Gy constraints for the rectum, mainly due to the small difference between the prescribed dose to the whole prostate and the dose to the DIL.

The number and total volume of DILs combined with the different prescribed doses did not significantly influence the OARs. The only variable that had a significant effect on dose constraints was the location of the DIL, a finding that is consistent with those reported by Kim et al. (5). Those authors compared three plans for localized PCa with dominant lesions located in posterior segments, all with 35 Gy (5 fx) to the whole prostate with varying doses to the DILs, as follows: 1) 35 Gy + no boost, 2) 35 Gy + 35 Gy boost, and 3) 35 Gy + 45 Gy boost. Based on the results, Kim and colleagues concluded that a moderate dose escalation of 40 Gy in 5 fractions should be appropriate. Given our results, we assume that for small focal lesions with an anterior localization, the dose could be even higher than 45 Gy, possibly up to 47.5Gy.

Due to the complexity of these plans different dosing methods, locations, and arrangement of the DILs it is important not only to ensure the correct planning strategy, but also to check the accuracy of the dose distribution calculations and the feasibility of delivering these plans on the linear accelerator. For this reason, we verified all calculations (DoseCHECK module), performed dosimetric pre-verification (2D-EPID-based – fraction 0, OCTAVIUS-4D Rotational Phantom with the OCTAVIUS Detector 1600 SRS), and verified the dose distributions in the phantom model using radiochromic film dosimetry and transit dosimetry. In contrast to other studies where only SBRT-SIB planning dosimetry parameters were examined (5).

Our findings demonstrate that transit and non-transit 2D-EPID-based dosimetry show agreement above the assumed criteria (96% passing rate for the non-transit and 92% for transit dosimetry) (**Figures 4**–**8**). Comparing 3D dose distributions reconstructed with OCTAVIOUS-4D and non-transit 2D-EPID dosimetry showed global DD3% and DTA 2mm gamma passing rates of 99% and 99.2%, respectively, similar to the findings described by Olaciregui-Ruiz et al. (29). As shown in **Figures 6**, 7, non-transit OCTAVIOUS-4D dosimetry slightly outperforms non-transit PerFRACTION Fraction 0 (**Figures 4**, **5**). According to criteria in the AAPM TG-218 report, the tolerance limit should be set at 95% of points passing the global approach DD 3%, DTA 2mm, TH 10% gamma analysis (23). All of our results met these limitations, both for OCTAVIOUS-4D with 1600 SRS array dedicated equipment to SBRT treatment and commercially available web-based platform as an element of Suncheck platform perFRACTION (30). In terms of the transit dosimetry results, taking into account the inhomogeneities in the anthropomorphic phantom (**Figures 4**, 5), we observed good agreement with non-transit 2D-EPID dosimetry and OCTAVIOUS-4D, which means both verification devices are acceptable. The PerFRACTION module is fully automated in our clinic.

This study also demonstrates good agreement between 2D-EPID-based, pre-treatment, and in vivo transit dosimetry using a commercially available automated system, in line with the results reported in the large cohort study by Bossuyt et al. (31).

The 3D anthropomorphic phantom in this study included inserts for radiochromic films to perform transit dosimetry (using another tool). The gamma methods results were also very promising (> 92%); additionally, we examined plans in high dose regions of the prostate PTV results for film 1 (**Figure 8**) and lower doses through bladder film 2 (**Figure 3**). These results support the measurements obtained with 2D-EPID-based transit dosimetry and are in line with the findings in the study by Giacometti et al. (28).

### Strengths and limitations

The main limitation of this study is that it was not possible to verify the dose escalation for DILs located in all 12 prostate segments due to the phantom design (**Figure 1**). By contrast, the main strength of this study is that it demonstrates that it is possible to treat DILs with dose-escalated SIB in various localizations in the prostate gland, including those located in posterior segments, with clinically acceptable PTV coverage within the normal dose constraints for OARs for dose up to 50 Gy. Another strength is that we performed dose delivery verification at two time points in the radiotherapy process, before (non-transit dosimetry) and during (transit dosimetry) treatment.

## Conclusion

The results of this study, conducted in a phantom model of prostate cancer, show that moderate dose escalation up to 45 Gy appears to be appropriate for the delivery of a simultaneous integrated boost to the dominant intraprostatic lesions located in posterior segments of the prostate gland. Similarly, the same dose limit applies in cases with ≥ 3 DILs located in other areas of the prostate gland. The dosimetric results for all plans verified by transit and non-transit 2D-EPID dosimetry (SunCheck) for SBRT-SIB were equivalent to those obtained by conventional detector arrays (OCTAVIOUS-4D) and radiochromic films for patient-specific quality assurance.

## Data availability statement

The original contributions presented in the study are included in the article/supplementary material. Further inquiries can be directed to the corresponding author.

## Author contributions

AS, MK-M, KG, AR, JM contributed to the conception and design of the study. Methodology: AS, MK-M. AS organized the database. AS, MK-M, KG performed the statistical analysis. AS wrote the first draft of the manuscript. AS, MK-M, KG wrote sections of the manuscript. All authors contributed to the article and approved the submitted version.
